# Foam formation during drainage of a surfactant solution in a microfluidic porous medium model

**DOI:** 10.1038/s41598-023-48442-5

**Published:** 2023-12-09

**Authors:** Nicolle Lima, Shima Parsa, Sidnei Paciornik, Marcio S. Carvalho

**Affiliations:** 1 Department of Mechanical Engineering, Pontifícia Universidade Católica do Rio de Janeiro, Rio de Janeiro, Brazil; 2 School of Physics and Astronomy, Rochester Institute of Technology, Rochester, USA; 3 Department of Chemical and Materials Engineering, Pontifícia Universidade Católica do Rio de Janeiro, Rio de Janeiro, Brazil

**Keywords:** Chemical engineering, Fluid dynamics, Surfaces, interfaces and thin films

## Abstract

Foam has been shown to have great potential to significantly improve sweep efficiency during gas injection in oil recovery, remediation of contaminated sites, gas storage, and acidification processes. The gas mobility reduction largely depends on the generation and stability of lamellae in the pore space that traps the gas phase. Most available analyses focus on foam formation during the co-injection of gas and liquid phases at different fractional flow (foam quality) or flow of foam formed before being injected in the porous media. During surfactant-alternating-gas (SAG) injection, foam is formed as the aqueous phase is displaced by the gas slug that follows. The dynamics of lamellae formation and their stability are different from that of a co-injection process, since the amount of surfactant available to stabilize the gas-liquid interfaces is fixed as fresh surfactant solution is not injected together with the gas phase. This work studies foam formation during the drainage of a surfactant solution by gas injection at a fixed flow rate. A transparent microfluidic model of a porous medium is used in order to enable the correlation of pore-scale phenomena and macroscopic flow behavior. The results show that the maximum number of lamellae increases with surfactant concentration, even much above the critical micelle concentration (CMC). The availability of surfactant molecules needed to stabilize newly formed gas-liquid interfaces rises with concentration. The higher number of lamellae formed at higher surfactant concentration leads to stronger mobility reduction of the gas phase and longer time needed for the gas to percolate through the porous medium.

## Introduction

Carbon dioxide, nitrogen, hydrocarbon gases and steam, which are examples of different gases employed in Enhanced Oil Recovery (EOR) methods, have very low viscosity when compared to the other fluids present in an oil reservoir. This high mobility ratio between the injected and displaced phases leads to unstable displacement front and inefficient sweep of the reservoir. The formation of preferential flow paths causes early breakthrough of the injected gas and low oil recovery factor. The phenomenon is amplified by gravity segregation, which occurs because of the relative low density of the gas, and reservoir heterogeneity.

The mobility of the injected gas phase and, consequently, the poor reservoir sweep efficiency, can be drastically reduced by injecting the gas phase in the form of foam^1–3^. Foam is a dispersion of a gas in a continuous liquid phase at which the gas bubbles are separated from each other by thin stable liquid films, called lamellae. The resistance to the motion of the many lamellae present in the foam structure through the pore space renders a high effective viscosity to the foam.

Foam can be pre-formed and injected or formed in situ by continuously co-injecting gas and surfactant solution or by surfactant-alternating-gas injection^4–7^. Foam can provide mobility control in systems with large permeability contrasts, leading to more uniform displacement^8,9^. Foam has been used not only in enhanced oil recovery but also in matrix-acidifying treatments^10^, in gas storage^11^, and in remediation of contaminated sites by containing the contaminant due a decreased permeability to water^12^.

Foam has still not evolved into a typical oil recovery technology for reservoir engineering despite its distinct features, wide range of applications, and years of development. The success of foam as a mobility control agent highly depends on the generation, propagation through the porous medium and stability of lamellae residing in the pores.

The macroscopic flow behavior of foam is directly related to its texture and structure. Foam formation is a complex phenomenon. Foam is formed during two-phase flow of gas and surfactant solution by different mechanisms: *lamella leave behind*, *lamella division*, *snap-off* and *pinch-off*^13–16^. Several experimental results indicate that foam is only formed above a minimum pressure gradient (or flow velocity)^13,17,18^. Most of these analyses were carried out by co-injecting the gas and liquid phases and have explored the effect of foam quality (fractional flow) and injection rate on foam mobility. The surfactant concentration in the aqueous phase is typically fixed, close to the critical micelle concentration (CMC) of the surfactant, since above this value the interfacial tension between both phases is constant. In surfactant-alternating-gas process (SAG), which involves injecting slugs of surfactant solution followed by gas, foam formation dynamics is different. Lamellae are formed during the drainage of the aqueous phase. The amount of surfactant available to stabilize newly formed lamellae is fixed, since there is no injection of a fresh aqueous phase together with the gas. Hence, the extent of lamellae formation and, therefore, of mobility control of the formed foam should vary with the surfactant concentration even for values above the CMC. To the best of our knowledge, this effect has not been studied before.

The development of accurate multi-scale flow models of foam in porous media requires fundamental knowledge of the relationship between pore-scale phenomena and macroscopic behavior. Recent improvements in the fabrication techniques of microfluidic devices have led to a growth in the use of flow visualization at the pore scale to gain detailed information on two-phase flow in porous media^19,20^. Pore scale visualization has greatly advanced the fundamental understanding of flow of injected foam through porous media^21–25^. However, most of the available studies focus on the flow of injected foams, not on the foam formation process that occurs as a surfactant solution is displaced by a gas phase.

This work analyzes the dynamics of foam formation during the displacement of surfactant solutions by gas injection at a constant volumetric flow rate in a porous medium, which is directly related to foam formation in SAG oil recovery process. The experiments were performed in a transparent porous medium microfluidic model, which enabled the visualization of pore-scale phenomena together with measurements of macroscopic flow behavior. The different lamellae formation mechanisms were visualized and foam characteristics were quantified by the time-evolution of the number of lamellae per unit area and pressure difference. The effect of surfactant concentration on the process dynamics was studied up to concentrations much higher than the CMC.

## Materials and methods

The dynamics of foam formation in the pore space of a microfluidic porous medium model during the displacement of surfactant solution by injected gas (air) at a fixed volumetric flow rate was analyzed. The evolution of the foam structure and the inverse of gas mobility (apparent gas viscosity) were evaluated as a function of the surfactant concentration.

### Microfluidic micromodel

**Figure 1:**
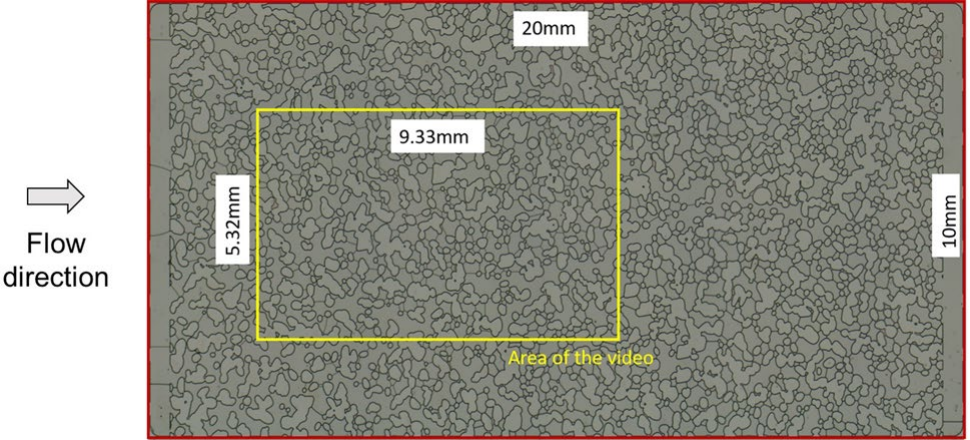
Image of the porous media micromodel used in the study. The area marked in yellow represents the region at which the evolution of the number of lamellae was quantified during foam formation.

The fluid injection experiments were performed on a microfluidic porous medium model made of borosilicate glass, produced by Micronit. The micromodel is water-wet and has a porous matrix 20 mm long × 10 mm wide and 20 μm etching-depth. The inlet and outlet flow distribution chambers are 500 μm wide. The device was designed by randomly placing rock-grain shaped structures to resemble the actual geometry of a slice of a sandstone rock. Pore bodies and throats appear between the solid matrix structures. The approximate size of the smallest throat is 12 μm, while the largest throat is approximately 250 μm. The pore volume is 2.3 μL, which corresponds to a porosity of 0.57. The permeability of the model, according to the supplier, is 2.5 D. The porosity and absolute permeability values are higher than typical reservoir rocks, however working with a transparent 2D geometry allows the visualization of pore scale events and the correlation between these events to macroscopic flow behavior. Figure 1 shows an image of the micromodel. The evolution of phase distribution inside an area of 9.33 × 5.32 mm^2^ of the micromodel, marked in yellow in the figure, was recorded during the entire duration of the experiments in order to evaluate the evolution of foam texture and quantify the number of lamellae in the pore space. After reaching steady state, an image of the entire pore space was acquired.

### Experimental setup and procedure

**Figure 2:**
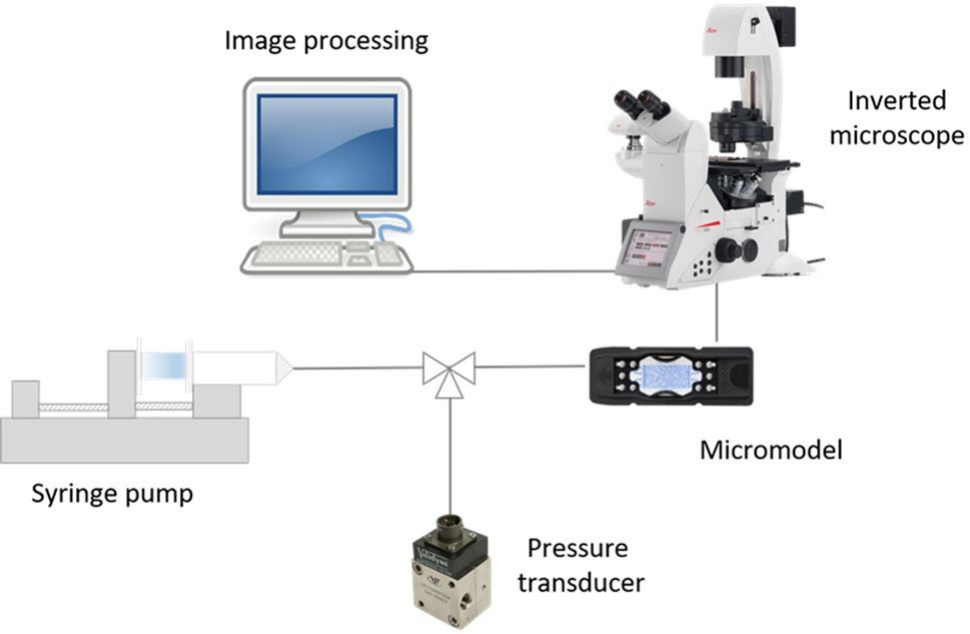
Experimental setup.

The experimental setup is sketched in Fig. 2. Both the aqueous and gas phases were injected using a syringe pump (Harvard Apparatus) with gastight glass syringes (Hamilton), with a Teflon termination and Luer-Lock coupling. A three-way valve was used to connect the pressure transducer to the injection line. The microfluidic device was placed on the stage of an inverted microscope (Leica DMi8) for visualization. Leica’s MC170 HD camera was used to record the evolution of the gas injection and lamellae formation during each experiment.

The differential pressure was measured using a DP15TL pressure transducer (Validyne) placed just upstream of the microfluidic device. The diaphragms used have 0.5% accuracy and pressure ranges of 0-5 psi and 0-20 psi. The outlet was open to atmosphere.

In order to ensure a complete saturation of the pore space with the aqueous phase without gas bubbles, the micromodel was first saturated with carbon dioxide. After this initial step, the model was fully saturated with the aqueous phase (water or surfactant solution).

The aqueous phase was displaced by gas injected at a constant volumetric flow rate of $q_g = 1$ mL/h until the differential pressure reached steady-state. The range of capillary numbers explored was $Ca = \mu _a V / \sigma = 2.1 \times 10^{-5}$ to $4.1 \times 10^{-5}$. The capillary number is defined in terms of the aqueous phase viscosity $\mu _a$, the interfacial tension between the phases $\sigma$ and the Darcy velocity *V*.

Sodium dodecyl sulfate (SDS) was the surfactant used in the experiments. The solution was prepared by dissolving the powder surfactant in deionized water, filtrated through a 0.45μm filter. Aqueous dye was added to the surfactant solution to better distinguish liquid from other fluids and glass matrix in the visualization experiments.

Surface tension measurements were carried out in aqueous solutions of SDS in order to determine the critical micellar concentration (CMC) of the system. All measurements were done on a DCAT25 tensiometer by DataPhysics Instruments using a Wilhelmy plate. The reported values of the surface tension were obtained at a constant temperature of 23 °C. The equilibrium surface tension of water with red dye used for the preparation of solutions was 61.6 mN/m. The interfacial tension value stabilizes at 34.4 mN/m at high enough surfactant concentration. The measured critical micelle concentration (CMC) was approximately 3 g/L.

### Quantification by image analysis

The images recorded during each experiment were processed using Fiji (Fiji Is Just ImageJ)^26^, which has many built-in plugins that facilitate scientific image analysis. The main goal of the image analysis was to evaluate the remaining aqueous phase and to determine the evolution of the number of lamellae.

First, an image of the device completely saturated with air was used to define the configuration of the solid matrix and pore space. This image is referred to as *Mask*.

**Figure 3:**
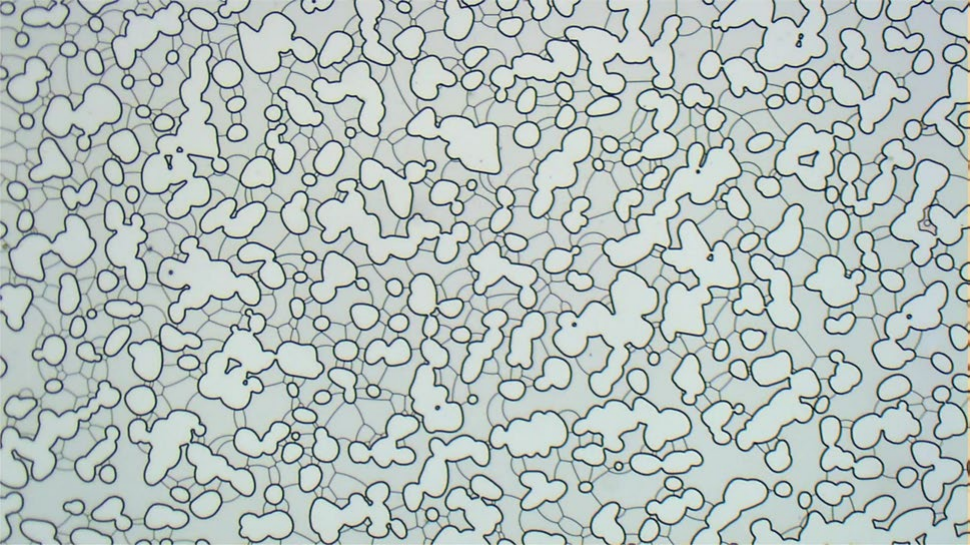
Porous medium after displacement of surfactant solution by air. Pore space presents several lamellae.

During the surfactant solution displacement by gas injection, frames obtained every 10 seconds from the recorded video were analyzed. Figure 3 presents an example of such images. The presence of liquid films defining multiple gas bubbles is clear.

**Figure 4:**
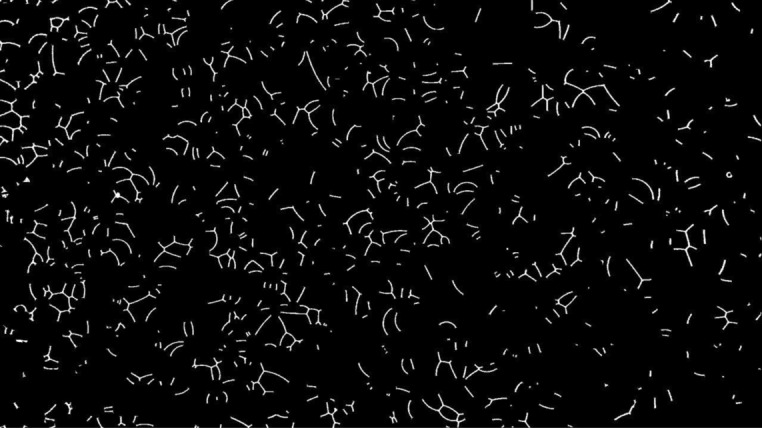
Cleared image containing the lamellae.

A plugin called bUnwarpJ was used to align the images of each time step and the *Mask*, which is crucial for image subtraction operations that were used to count the number of lamellae. bUnwarpJ is an algorithm for elastic and consistent image registration^27^. The Fiji macro commands used for this operation were:
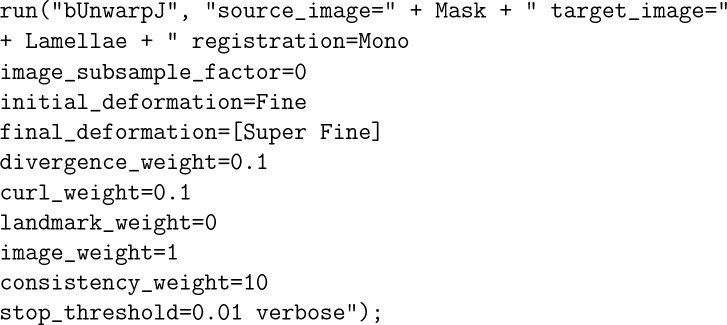


The next step was the binarization of both images. Three threshold algorithms were used, Isodata^28^, Huang^29^ and Triangle^30^, depending on the illumination condition of each experiment. Spurious isolated objects (smaller than 100 px^2^) that could be dirt in the glass device or impurities in the fluids were removed after the binarization. The Fiji macro commands used for these operations were:
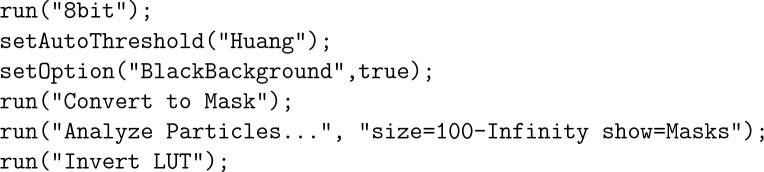


To isolate the lamellae and eliminate the solid matrix boundaries from the image, a logical operation (AND) was conducted between the binary and aligned images of each time step and the inverted *Mask*. The Fiji macro command used for this operation were:
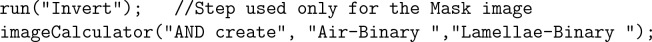


Because of small differences on the representation of the solid grain boundaries in the two images, the subtraction operation is not perfect and generates very small objects. Objects smaller than 10 px^2^ were removed using the commands:
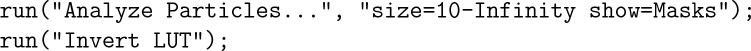


The result of these operations is shown in 4. The number of lamellae is not equal to the number of isolated objects in Fig. 4 since different lamellae may be connected forming a single object. To isolate each lamella, the objects are skeletonized, which involves repeatedly removing pixels from the borders of objects until they are reduced to single-pixel-wide shapes. A plugin Analyze Skeleton 2D/3D is used to sort the branch, node and endpoint of each object and represent them with different colors^31^. The nodes (connection between different branches) have a tone below 71, so they can be eliminated by a treshold operation. The Fiji macro commands used for these operations were:

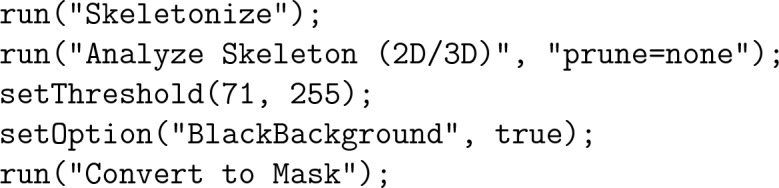



**Figure 5:**
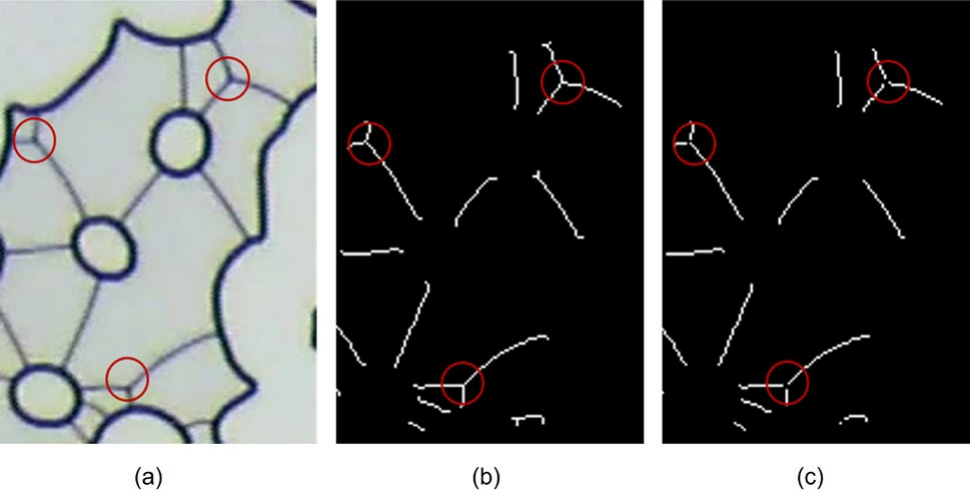
(**a**) original image, nodes are marked by a red circle, (**b**) nodes identified in the skeletonized image, (**c**) image with nodes removed.

The result of these operations is exemplified in Fig. 5, which shows (a) the original image, containing the solid grain boundaries and lamellae, (b) the skeletonized image after subtracting the *Mask* and (c) image at which the nodes where removed and lamellae are not connected to each other.

After the series of image operations described before, the number of lamellae is equal to the number of isolated objects in the image. During the quantification of the number of lamellae, only objects larger than 15 px^2^ ($\approx 18.2^2 \mu m^2$) are considered. The number of lamellae in the image is determined by the command:
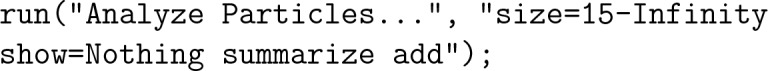


The previous groups of macro commands were put together in a single macro that could be run automatically and reproducibly. The macro took as input the reference frame and iterated over a sequence of frames containing lamellae, giving as output the number of lamellae for each frame.

## Results and discussion

The dynamics of the aqueous phase displacement flow by gas injection at a constant flow rate can be characterized by the evolution of the gas phase mobility $\lambda _g$, which can be evaluated directly from the measurement of the pressure difference $\Delta P(t)$ using Darcy equation:

$$\begin{aligned} v_g = \lambda _g K \frac{\Delta P(t)}{L}. \end{aligned}$$

$v_g = q_g / A$ is the gas phase Darcy velocity, *K* is the absolute permeability of the micromodel and *L* is the length of the porous medium. The inverse of the gas phase mobility can be interpreted as an apparent gas viscosity.

**Figure 6:**
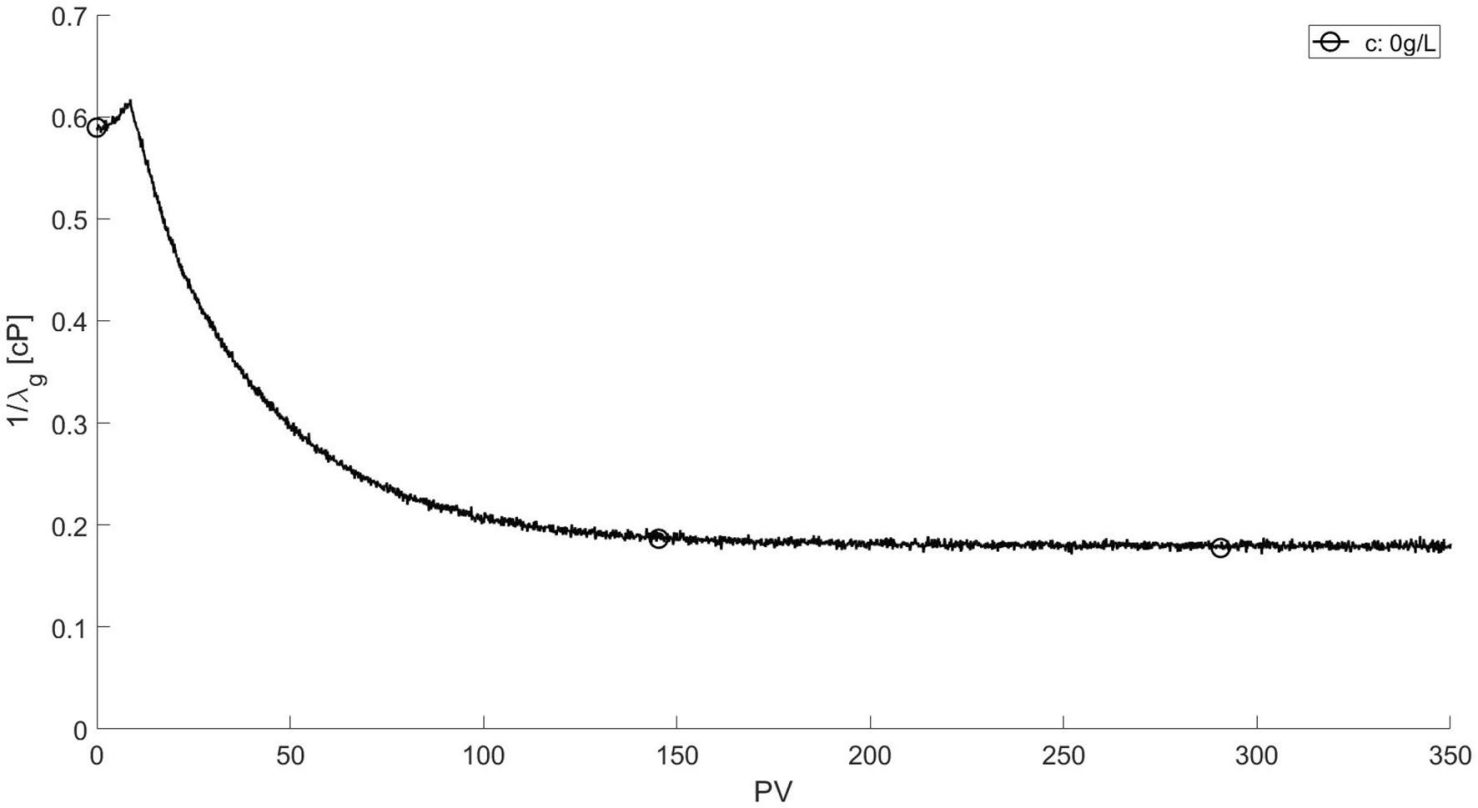
Evolution of the inverse of gas mobility $1 / \lambda _g$ during water displacement by gas injection at $q_g = 1$ mL/h.

**Figure 7:**
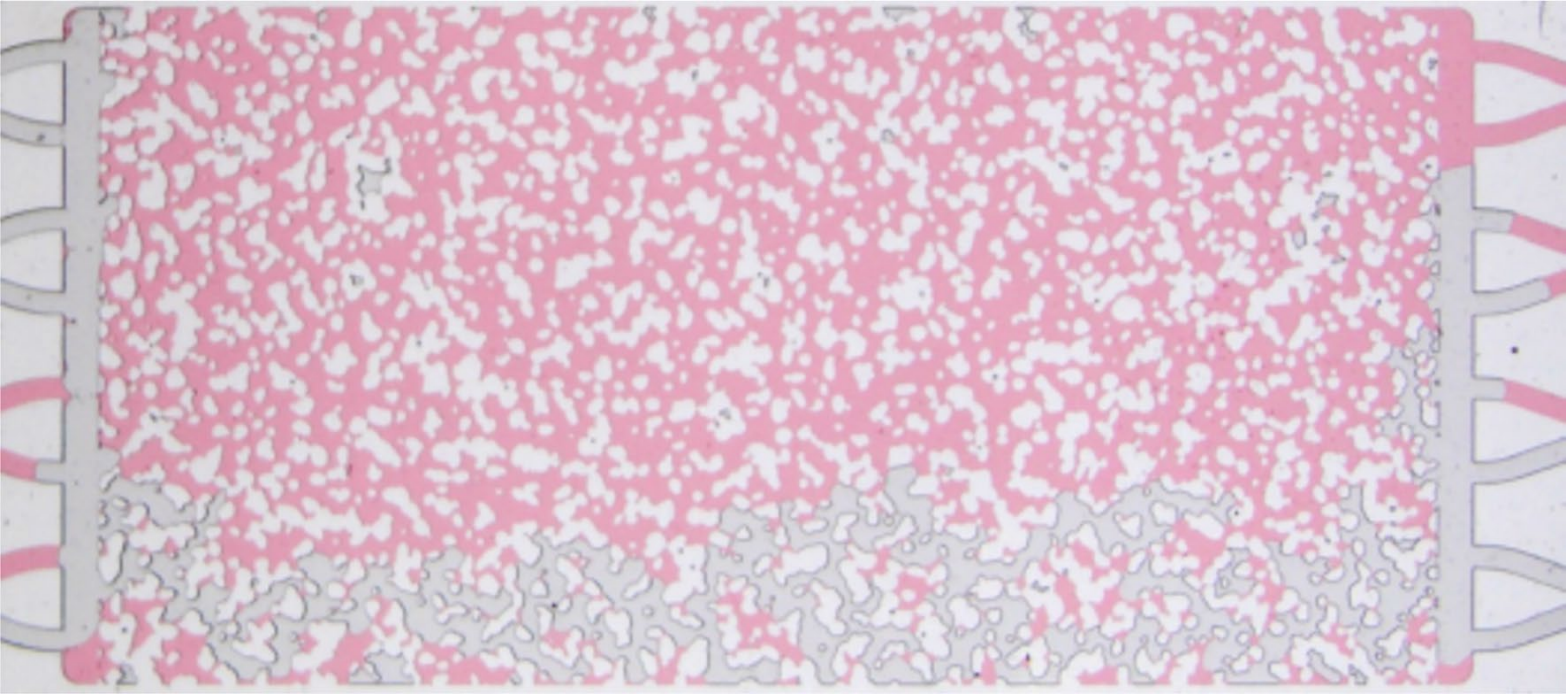
Phase distribution at steady state after water displacement by gas injection at $q_g = 1$ mL/h. Water is dyed in red. The remaining water saturation is 70 %.

The displacement of pure water ($c_s = 0$ g/L) is used as a base case. The evolution of the inverse of the gas mobility $1 / \lambda _g$ is presented in Fig. 6; the corresponding capillary number is $Ca = 2.1 \times 10^{-5}$. The apparent gas viscosity slightly rises in the initial stages of the displacement process and then falls as the lower viscosity phase (gas) displaces the higher viscosity phase (water) and gas saturation in the pore space increases. The inverse of the mobility reaches steady state of approximately $1/\lambda _g \approx 0.18$ cP after 28 minutes (200 pore volumes). The phase distribution after steady state is presented in Fig. 7. The water phase is dyed in red. The injected gas forms a preferential path that percolates the porous material due to the high viscosity ratio between the phases. The amount of water remaining in the micromodel after gas injection was approximately 70%. Because the water phase had no surfactant, foam was not formed during the displacement process of the aqueous phase.

**Figure 8:**
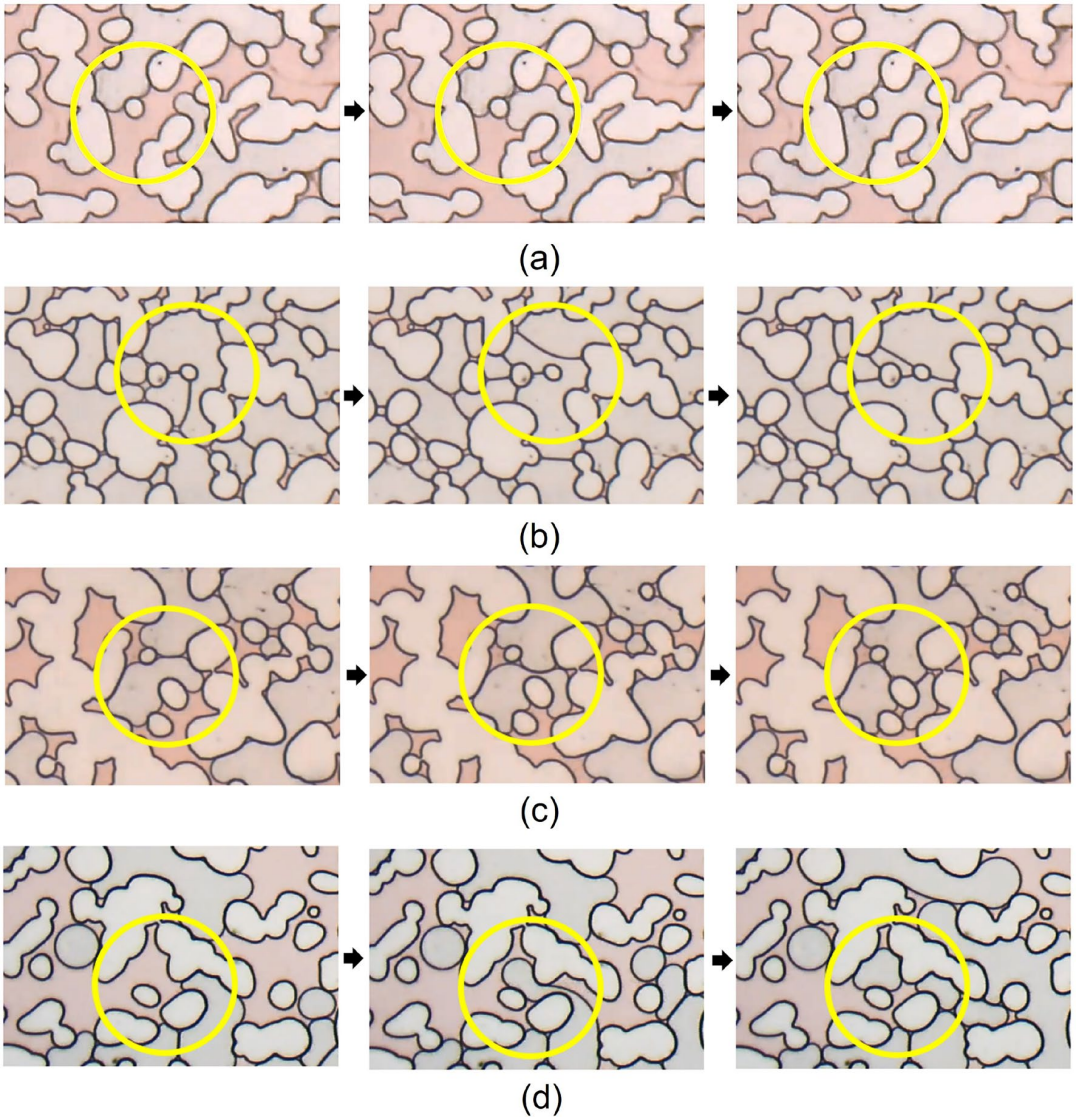
Visualization of different mechanisms of lamellae formation: (**a**) leave behind, (**b**) lamella division, (**c**) pinch-off, and (**d**) snap-off.

When surfactant is present in the aqueous phase, stable thin films of liquid are formed as the gas phase displaces the aqueous phase. Figure 8 presents a sequence of snapshots illustrating lamellae formation by the different mechanisms discussed in the literature^13–16^: (a) leave behind, (b) lamella division, (c) pinch-off, and (d) snap-off. As more lamellae are formed, the mobility of the gas phase is reduced, which leads to higher injection pressure.

**Figure 9:**
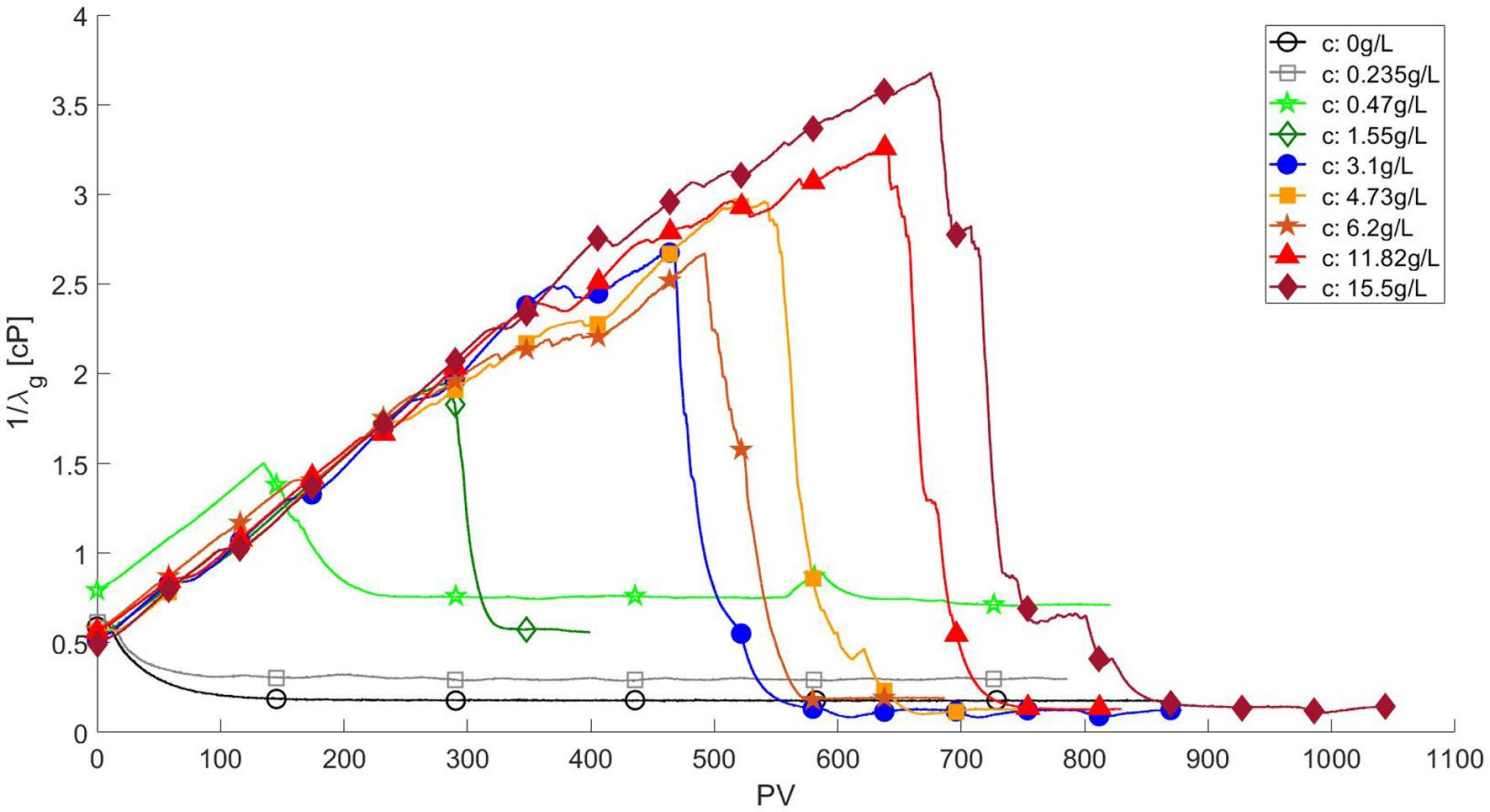
Evolution of the inverse of gas mobility $1 / \lambda _g$ during the displacement of surfactant solution with different concentrations by gas injection at $q_g = 1$ mL/h. The response for water displacement is shown as a reference. Cases with surfactant concentration below CMC are marked by open symbols, whereas the cases with surfactant concentration above CMC are marked by closed symbols.

The surfactant solution displacement by gas injection was studied for a range of surfactant concentration from $c_s = 0.235$ to $c_s = 15.5$ g/L, which corresponds to 5.2 $\times$ CMC. Figure 9 presents the time evolution of the inverse of gas mobility $1 / \lambda _g$ as a function of surfactant concentration, including the pure water displacement case shown before ($c_s = 0$), as a basis for comparison. The evolution for the cases with surfactant concentration below CMC are marked by open symbols, whereas the cases with surfactant concentration above CMC are marked by closed symbols in the plot.

**Figure 10:**
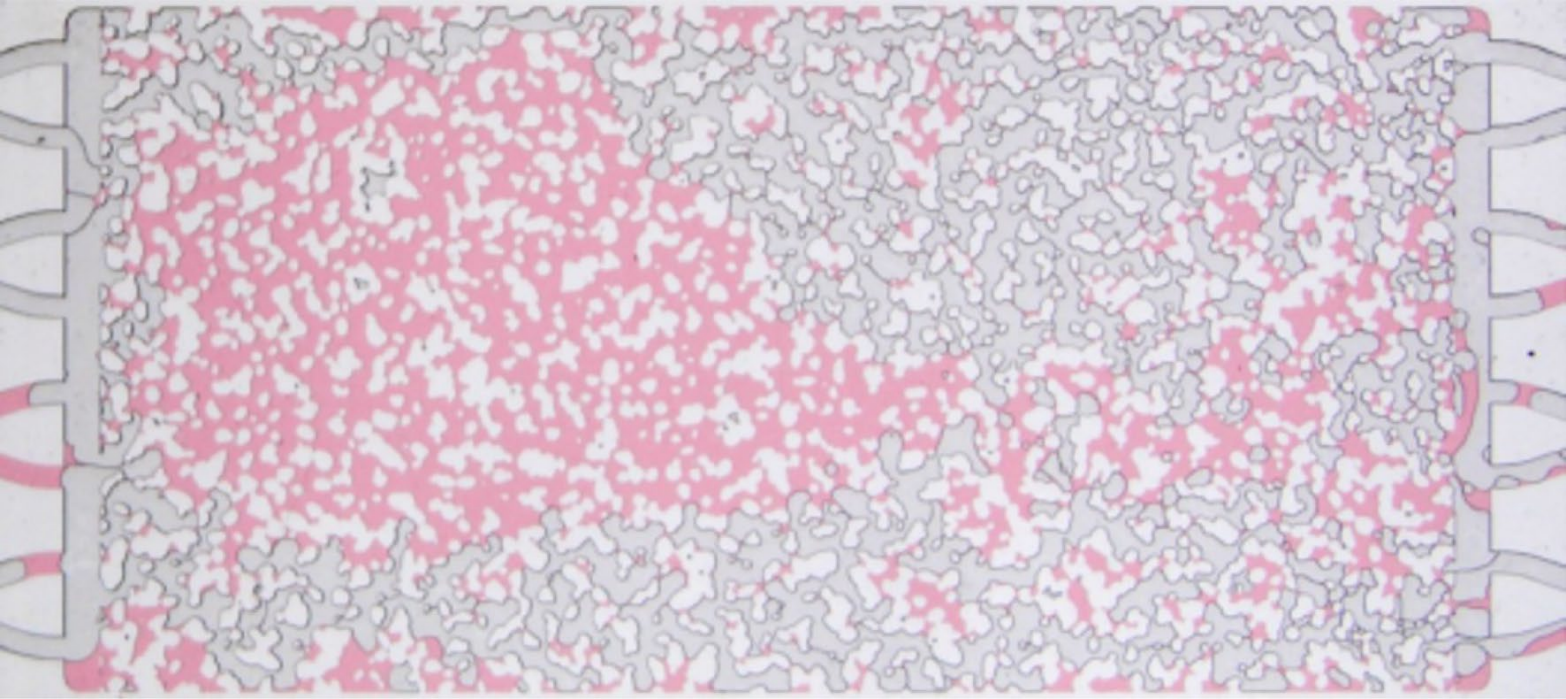
Phase distribution at steady state after surfactant solution ($c_s = 0.235$ g/L) displacement by gas injection at $q_g = 1$ mL/h. Water is dyed in red. The remaining water saturation is 45 %.

At the lowest surfactant concentration explored, $c_s = 0.235$ g/L, the apparent gas viscosity drops quickly as in the case without surfactant, but it stabilizes at a slightly higher value, $1/\lambda _g \approx 0.3$ cP. The higher apparent gas viscosity can be associated with the added resistance of the gas flow due to the lamellae that were formed as the surfactant solution is displaced by the injected gas phase. The added gas flow resistance leads to flow diversion; the phase distribution at steady state, presented in Fig. 10, clearly shows two preferential gas flow paths, connecting the inlet and outlet chambers. The remaining aqueous phase saturation is approximately 0.45, much lower than that observed in the pure water displacement experiment. It is important to note that the inverse of the gas mobility is higher in the displacement of the surfactant solution despite the higher gas saturation. Most of the gas paths are blocked by lamellae and part of the gas phase present in the pore space is trapped.

**Figure 11:**
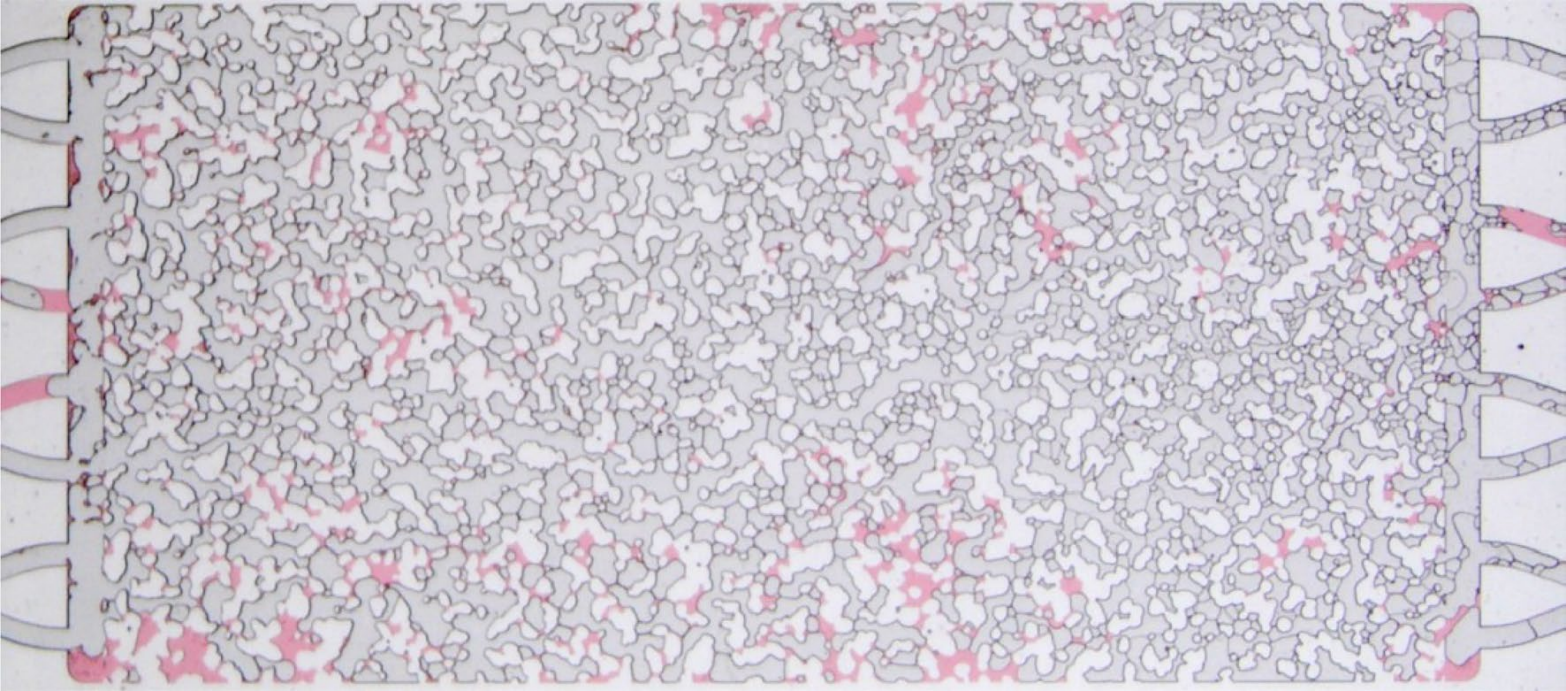
Phase distribution at steady state after surfactant solution ($c_s = 0.47$ g/L) displacement by gas injection at $q_g = 1$ mL/h. Water is dyed in red. The remaining water saturation is 8 %.

**Figure 12:**
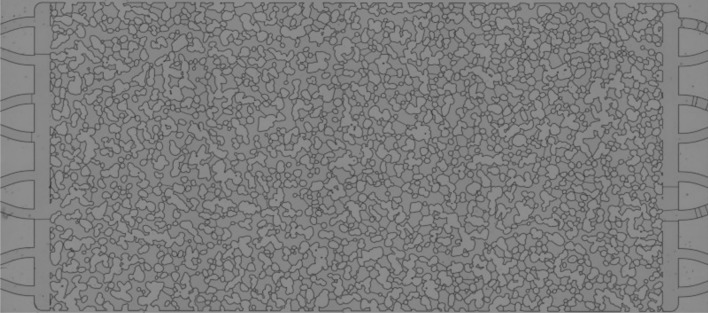
Phase distribution at steady state after surfactant solution ($c_s = 1.55$ g/L) displacement by gas injection at $q_g = 1$ mL/h. Aqueous phase is only present as thin films.

Higher surfactant concentration in the aqueous phase drastically changes the displacement flow behavior. Even at low concentration, e.g. $c_s = 0.47$ g/L $\approx 0.2 \times$ CMC, the mobility of the gas phase is drastically reduced by the formation of stable lamellae leading to higher injection pressure and consequently higher apparent gas viscosity, which rises for approximately 20 minutes (145 pore volumes) and reaches a maximum value of $1/\lambda _g \approx 1.5$ cP. After this, the apparent gas viscosity drops to a steady state plateau as the gas creates a percolated paths connecting the inlet and outlet. The phase distribution at steady state is shown in Fig. 11. The remaining water (marked in red) saturation is approximately 8%, much lower than that observed with pure water (70%) and at $c_s = 0.235$ g/L (45%). A large number of lamellae can be observed, mainly in the downstream region of the microfluidic device. The presence of a large number of lamellae increased the resistance to gas flow (higher apparent viscsoity), led to flow diversion and an efficient displacement of the aqueous phase. At a higher surfactant concentration but still below the CMC, e.g., $c_s = 1.55$ g/L $\approx 0.52 \times$ CMC, the maximum apparent gas viscosity rises to $1/\lambda _g \approx 2$ cP. The mobility of the gas phase during the aqueous phase displacement is low enough that, at steady state, water is only present in the pore space as thin stable films (lamellae), as it is clear in Fig. 12.

The maximum apparent gas viscosity continues to rise with surfactant concentration, even above the CMC; it reaches $1/\lambda _g \approx 3.7$ cP for the highest surfactant concentration explored, $c_s = 15.5$ g/L $\approx 5.2 \times$ CMC. The number of injected pore volumes at which the maximum apparent viscosity is reached also increases with surfactant concentration.

**Figure 13:**
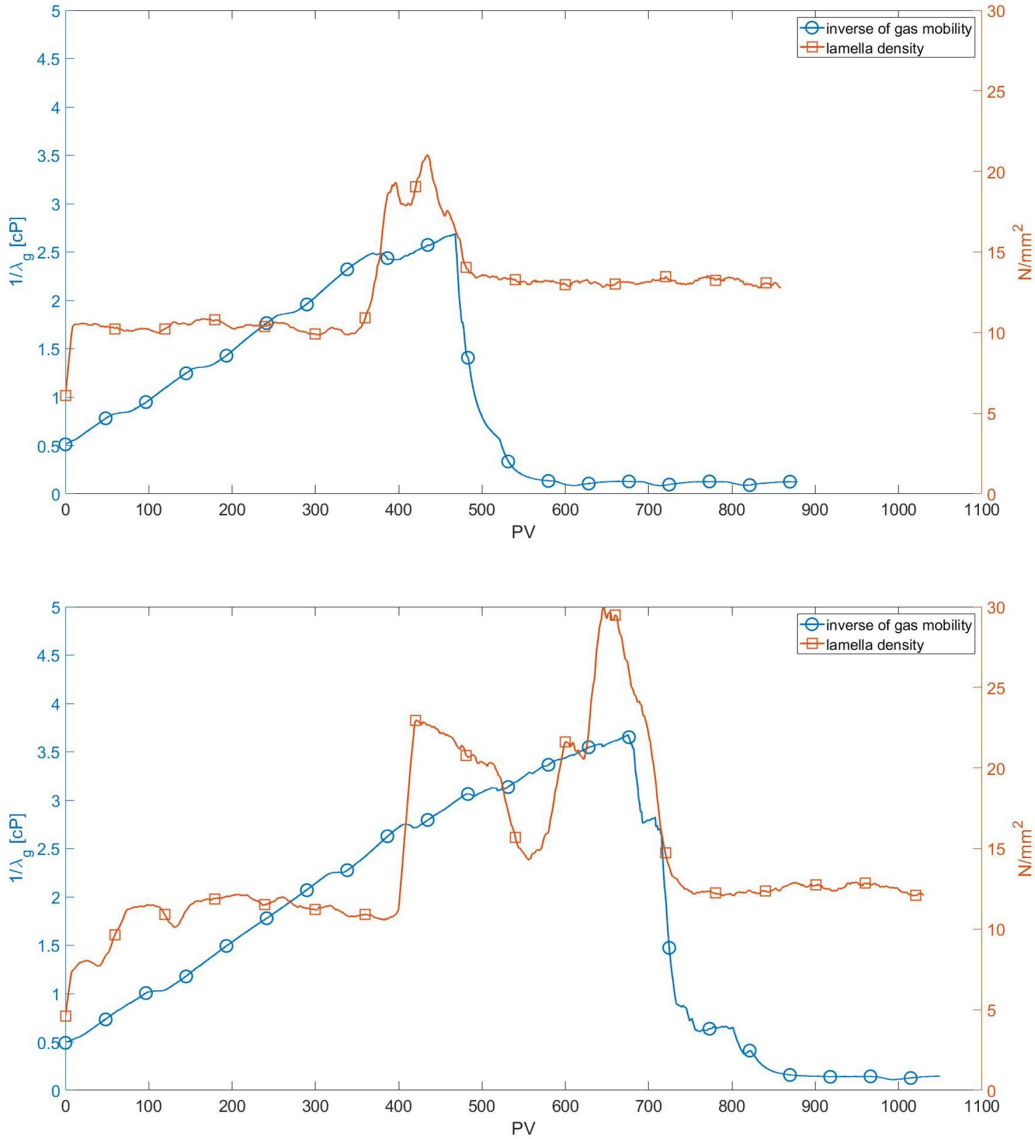
Evolution of lamella density and inverse of gas mobility: (**a**) $c_s = 3.1$ g/L ($\approx 1 \times$ CMC); (**b**) $c_s = 15.5$ g/L (5.2 $\times$ CMC).

As discussed before, the mobility of the gas phase, which is represented here as an apparent gas viscosity, is directly related to the number of lamellae occupying the pore space that trap the gas phase. Stronger foam with a large number of lamellae, and consequently a higher volume of trapped gas, has a higher flow resistance that leads to higher apparent viscosity. The evolution of the lamella density (number of lamellae per unit area) for $c_s = 3.1$ g/L ($\approx 1 \times$ CMC) and $c_s = 15.5$ g/L (5.2 × CMC) in a 5.3 × 9.3 mm^2^ area near the middle of the porous medium (marked in Fig. 1) are presented in Fig. 13. The evolution of the apparent gas viscosity is also presented in the plots. The rate of lamella formation in the initial stages is very high; a plateau is reached after a very short time. The evolution of lamella density occurs in spurts, with long periods of almost constant values followed by steep positive and negative gradients. This intermittency on the flow of foam through porous media has been observed and discussed in the literature^24,32,33^.

**Figure 14:**
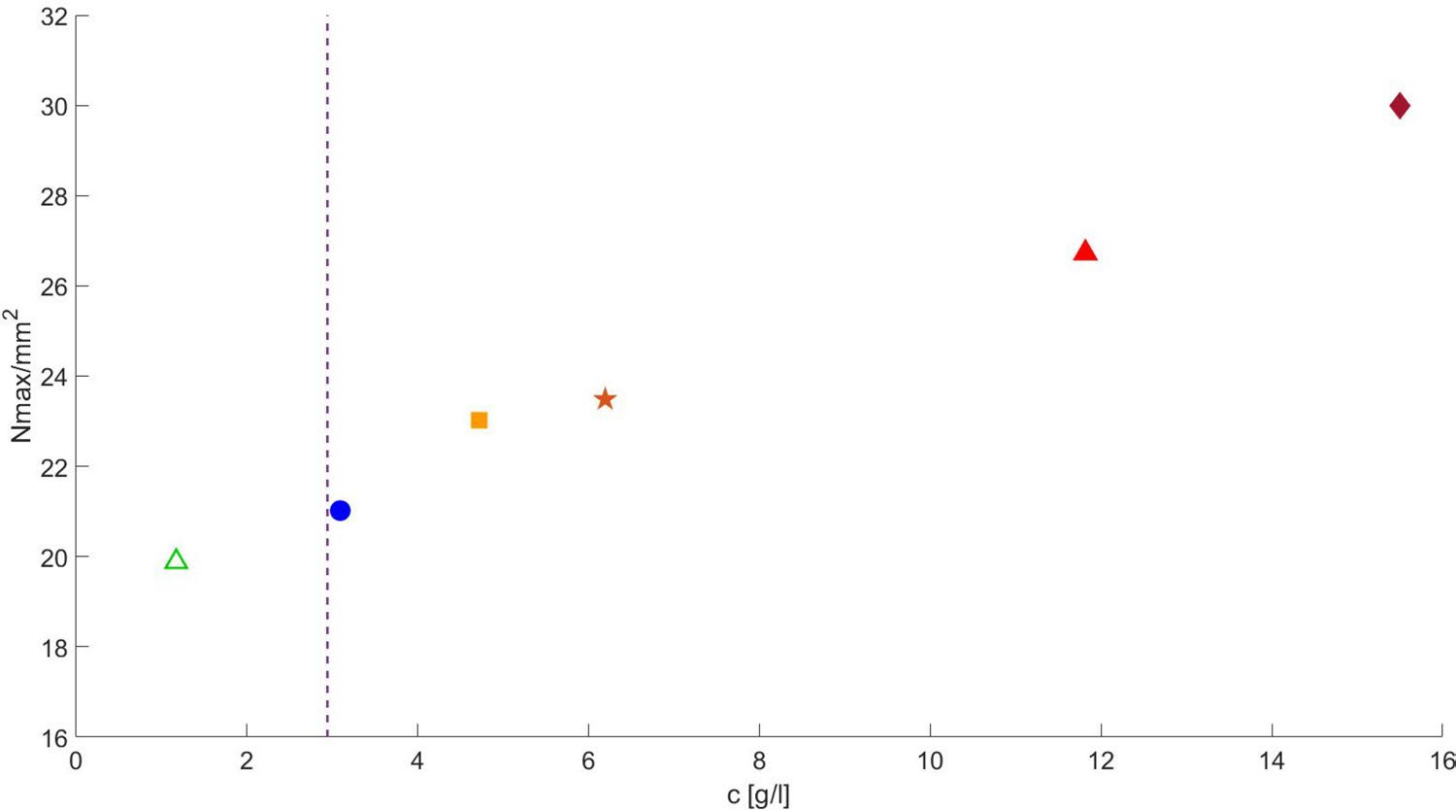
Maximum lamella density as a function of surfactant concentration. The CMC is $c_s = 3$ g/L, marked by the dotted vertical line.

The higher apparent viscosity occurs simultaneously with the maximum value of lamella density. The apparent viscosity drop approaching the steady state value, which indicates a percolated path being formed, occurs as the lamella density drops abruptly. The maximum number of lamellae rises with surfactant concentration, even above the CMC value, as presented in Fig. 14. It is important to note that the number of surfactant molecules available to stabilize the newly formed gas-liquid is fixed, since only gas is injected. Therefore, even though the interfacial tension value is constant above CMC, the higher the surfactant concentration, the more surfactant molecules are available to stabilize new lamellae that are formed during the displacement flow. This implies that at higher surfactant concentration, a larger number of lamellae can be stabilized, which is directly related to the continuous rise of apparent gas viscosity with surfactant concentration, even above the CMC value.

It is important to note that the apparent gas viscosity at the end of gas injection varies with the surfactant solution concentration, as it is clear from Fig. 9. In the displacement of pure water, the steady-state apparent gas viscosity (inverse of the gas mobility) is $1/\lambda _g \approx 0.2$ cP. The steady-state apparent viscosity rises with surfactant concentration for values below CMC, reaching $1/\lambda _g \approx 0.6$ at $c_s = 1.55$ g/L ($\approx 0.5 \times$ CMC). At surfactant concentration above the CMC, the apparent gas viscosity at the end of gas injection is lower, close to the value of the water displacement flow, e.g. $1/\lambda _g \approx 0.2$ cP. Most likely, the gas mobility at steady state is directly associated with the pore volume (mobile gas saturation) of the flow paths that fully connect the inlet and outlet chambers of the micromodel. The resolution of the images of the entire pore space did not allow this quantification and this behavior is still and under investigation.

## Final remarks

Foam formation during drainage of surfactant solution by gas injection at a fixed flow rate in a transparent microfluidic porous media model was analyzed. This flow resembles the process that occurs in surfactant-alternating-gas (SAG) injection. The formed foam was characterized by the evolution of the apparent gas viscosity (inverse of gas mobility) and lamella density in the pore space, which enabled the correlation of pore-scale phenomena to macroscopic flow behavior.

As the gas displaces the surfactant solution, flow visualization revealed that stable lamellae are formed by different mechanisms. The mobility of the gas phase falls, leading to an increasing apparent viscosity. The gas apparent viscosity continuously rises until reaching a maximum value, after which it abruptly falls. The value of the maximum apparent viscosity and the time at which it occurs rises with surfactant concentration, even above the CMC. For the highest surfactant concentration explored, approximately $5 \times$ CMC, the apparent gas viscosity was $1/\lambda _g \approx 3.7$ cP, close to $10 \times$ higher than the value observed in the flow of gas displacing an aqueous phase without surfactant.

The structure of the foam in an observation window that occupied close to 1/4 of the pore space was analyzed by image processing. The evolution of the number of lamellae occurs in spurts, with long periods of almost constant values followed by abrupt increase or decrease. The time at which the number of lamellae is maximum is approximately the same as the time at which the apparent viscosity is maximum.

## Data Availability

The datasets generated and analyzed during the current study available from the corresponding author on reasonable request.
